# Trajectories of risky drinking around the time of statutory retirement: a longitudinal latent class analysis

**DOI:** 10.1111/add.13811

**Published:** 2017-04-16

**Authors:** Jaana I. Halonen, Sari Stenholm, Anna Pulakka, Ichiro Kawachi, Ville Aalto, Jaana Pentti, Tea Lallukka, Marianna Virtanen, Jussi Vahtera, Mika Kivimäki

**Affiliations:** ^1^ Finnish Institute of Occupational HealthHelsinki/KuopioFinland; ^2^ University of Turku, Department of Public Healthand Turku University HospitalTurkuFinland; ^3^ University of TampereFaculty of Social Sciences (Health Science)TampereFinland; ^4^ Harvard T. H. Chan School of Public HealthBostonMassachusettsUSA; ^5^ Department of Public Health, Faculty of MedicineUniversity of HelsinkiHelsinkiFinland; ^6^ Department of Epidemiology and Public HealthUniversity College London Medical SchoolUK

**Keywords:** Ageing, alcohol, cohort, retirement, risky drinking, trajectory

## Abstract

**Background and Aims:**

Life transitions such as retirement may influence alcohol consumption, but only a few studies have described this using longitudinal data. We identified patterns and predictors of risky drinking around the time of retirement.

**Design:**

A cohort study assessing trajectories and predictors of risky drinking among employees entering statutory retirement between 2000 and 2011.

**Setting and Participants:**

A total of 5805 men and women from the Finnish Public Sector study who responded to questions on alcohol consumption one to three times prior to (w_−3_, w_−2_, w_−1_), and one to three times after (w_+1_, w_+2_, w_+3_) retirement.

**Measurements:**

We assessed trajectories of risky drinking (> 24 units per week among men, > 16 units among women, or an extreme drinking occasion during past year) from pre‐ to post‐retirement, as well as predictors of each alcohol consumption trajectory.

**Findings:**

Three trajectories were identified: sustained healthy drinking (81% of participants), temporary increase in risky drinking around retirement (12%) and slowly declining risky drinking after retirement (7%). The strongest pre‐retirement predictors for belonging to the group of temporary increase in risky drinking were current smoking [odds ratio (OR) = 3.90, 95% confidence interval (CI) = 2.70–5.64], male sex (OR = 2.77, 95% CI = 2.16–3.55), depression (OR = 1.44, 95% CI = 1.05–1.99) and work‐place in the metropolitan area (OR = 1.29, 95% CI = 1.00–1.66). Compared with the slowly declining risky drinking group, the temporary increase in risky drinking group was characterized by lower occupational status and education, and work‐place outside the metropolitan area.

**Conclusions:**

In Finland, approximately 12% of people who reach retirement age experience a temporary increase in alcohol consumption to risky levels, while approximately 7% experience a slow decline in risky levels of alcohol consumption. Male gender, smoking, being depressed and working in a metropolitan area are associated with increased likelihood of increased alcohol consumption.

## Introduction

Risky alcohol use is among the most significant contributors to the burden of disease world‐wide 1. Adverse life events, such as divorce and job loss, may increase alcohol consumption 2, 3. Retirement is a further significant life transition when substantial changes in daily life are experienced as retirees adapt to life without work. However, little is known about how risky alcohol consumption changes around the retirement transition.

Previous evidence on this issue is inconsistent 4. The Health and Retirement Study in the United States found an association between retirement and increased alcohol consumption 5, a finding replicated partially in a Dutch study, where the increase was observed only among participants whose retirement was involuntary 6. In other investigations, a study of Australian employees found no changes in alcohol consumption in any retirement group 7, and findings in a Norwegian study were mixed 8. In addition, both increasing and decreasing alcohol use trajectories have been reported in a cohort of elderly men and women in the United States 9, 10. Given this heterogeneity in findings, it is possible that there are multiple subgroups with different trajectories of alcohol consumption around retirement that are defined by different individual‐level factors 11.

A major limitation in most of the studies in this field of research is their reliance upon only two measurement points 6, 7, 8, making it impossible to distinguish short‐ and long‐term changes in alcohol consumption over time. A French GAZEL (*Gaz de France Electricité de France*) cohort assessed alcohol consumption annually from 5 years prior to retirement up to 5 years afterwards, and found the estimated prevalence of heavy drinking to be increased around the time of retirement, but that it decreased during the following 4 years 12. However, the low statutory retirement age (approximately 56 years) of the GAZEL cohort limits the generalizability of these results to other retiring populations.

To address some of these limitations, the aims of our longitudinal study were: (1) to identify trajectories of risky drinking using repeated survey waves around the time of retirement and (2) to assess pre‐retirement predictors of belonging to each trajectory. We focused on statutory retirees only, as the voluntary nature of retirement has been found to mediate the impact of retirement on health outcomes 6.

## Methods

### Design

This is a prospective cohort study of Finnish public‐sector employees with data collected both before and after the time of retirement, totalling four possible data waves per participant. Trajectories that describe how risky drinking changes over time were drawn using repeated questionnaire data on alcohol consumption for time‐periods covering transition to retirement. In addition to the changes in patterns of risky drinking, we assess pre‐retirement predictors of the risky drinking trajectories.

The Public Sector Employees' Pension Act in Finland set the statutory (or old age) retirement age as generally between 63 and 65 years until 2005, and between 63 and 68 years from 2005 onwards. Some individuals have kept their earlier retirement age from the previous pension act in which pension ages in some occupations were below 63 years (e.g. 60 years for primary school teachers, 58 for practical nurses).

### Study population

The study population consisted of participants of the Finnish Public Sector (FPS) study, a prospective occupational cohort study with identifiable questionnaire surveys. The eligible population of the original cohort included all employees who had been working for a minimum of 6 months in the target organizations, which included 10 towns and six hospital districts, between 1991 and 2005 (*n* = 151 901) 13. Nested survey cohorts included all participants who were employed by the participating organizations at the time of the surveys or had participated in a survey while employed, but later left the organizations (i.e. ‘leavers’). Surveys were repeated at 4‐year intervals and for this study we used data from repeated surveys performed for current employees in 2000–02, 2004 and 2008 and for leavers in 2005, 2009 and 2013. Data from the surveys were linked successfully to the records of all earnings‐related pensions for all participants through unique personal identification codes. Anonymized data without the identification codes were used for the analyses. The FPS study was approved by the Ethics Committee of the Hospital District of Helsinki and Uusimaa.

We centred the data around the actual retirement date of each individual. There were three possible study waves before retirement (w_−3_, w_−2_, w_−1_), and three possible waves after retirement (w_+1_, w_+2_, w_+3_). The difference between each wave was, on average, 4 years. Of all the FPS cohort members, we first identified those who were employed and responded to at least one survey in 2000–02, 2004 or 2008 (*n* = 81 587). Of these, 19 058 retired between 2000 and 2011. We included those who retired at their individual statutory retirement age if this was their first awarded pension scheme (*n* = 5893). Thus, those who were first granted disability or part‐time retirement were not included in our analyses. We further restricted the study population to those participants who had reported their alcohol consumption in surveys immediately before and after transition to statutory retirement (i.e. w_−1_ and w_+1_). This resulted in an analytical sample of 5805 people. Thus, depending on the retirement date, participants' observations came from one of the following alternative sets with a maximum of four waves: (1) w_−3_, w_−2_, w_‐1,_ w_+1_, (2) w_−2_, w_‐1,_ w_+1_, w_+2_ or (3) w_‐1,_ w_+1_, w_+2_, w_+3._ On average, these participants provided information on alcohol consumption at 3.7 (range = 2–4) of the possible four study waves during a follow‐up of 4–12 years.

### Assessment of retirement

All gainful employment in Finland is insured by a pension plan, which accrues pension. The Finnish Centre for Pensions coordinates all the earnings‐related pensions for permanent residents in Finland 14 and provided data on each participant's retirement. Pension start dates from 2000 to 2011 were obtained for all participants.

### Risky drinking

The participants reported their habitual frequency and amount of beer, wine and spirits consumption, in weekly units of alcohol. One unit is 12 g of alcohol, or the equivalent of a 33 cl bottle of beer, a 12 cl glass of wine or a 4 cl measure of spirits 15. The Finnish Ministry of Health and Social Affairs has set the lower limit for heavy use of alcohol to 24 and 16 units per week for men and women, respectively 16. Based on these national cut‐off points we determined heavy alcohol use as weekly alcohol consumption > 24 units for men and > 16 units for women (yes versus no). Some studies have used slightly lower cut‐offs for risky drinking, such as > 21 units per week for men and > 14 units per week for women 17, although the definition of a unit in studies from other countries can vary from 8 g to 14 g of pure alcohol. Others have defined heavy use, or binge drinking, as > 4 units per drinking occasion for women and > 5 units for men 18. In addition, we obtained information on extreme drinking occasions by asking the participants whether they had passed out at least once due to heavy drinking during the past 12 months (yes versus no) 19. Heavy alcohol use and extreme drinking occasions were not associated strongly, as suggested by a low Cramer's V value of 0.09. For the analyses, we defined risky drinking as reporting either heavy alcohol use or at least one extreme drinking occasion, or both.

### Covariates

Higher alcohol consumption has been associated with male sex 20, being married, having a higher educational level, having a higher income, being employed and being a smoker 18, 21. These were therefore treated as covariates. We obtained information from the employers' registers regarding participants' sex, age at retirement and occupational status categorized according to the Classification of Occupations by Statistics Finland 22, as in previous studies 23, 24, into three groups: ‘high’, including physicians and teachers; ‘intermediate’, including registered nurses and technicians; and ‘low’, including maintenance workers and cleaners. Level of education (high = university degree; intermediate = high school or vocational school; low = basic education) was obtained from Statistics Finland 25, while marital status (married/cohabiting; single; divorced/widowed), smoking (current smoker versus not), depression (‘Have you ever been diagnosed with depression?’ yes versus no) and self‐reported health (poor versus good) were elicited by the questionnaires. Data from the last measurement before retirement (w_−1_) were used in the regression analyses. As participants could enter the study (i.e*.* retire) during different calendar periods, a ‘cohort’ variable was created on the basis of the last data collection year before retirement (i.e. when w_−1_ is year 2000 then cohort = 1, when w_−1_ is year 2004 then cohort = 2, and when w_−1_ is year 2008 then cohort = 3). With this variable we could estimate possible effects due to general trends in per capita alcohol consumption. The area of the work‐place was examined by using a dummy variable based on whether the work‐place at w_−1_ was located in the metropolitan area versus other area.

Additional covariates possibly related to alcohol use included: job strain (i.e. high job demands and low job control, based on questions from the Job Content Questionnaire), body mass index (measured as self‐reported weight/height in metres squared, kg/m^2^, categorized as < 25: normal weight; 25–29.9: overweight; ≥ 30 obese 26) and physical activity (measured as weekly Metabolic Equivalent Task (MET) hours 27, categorized as < 14 MET hours/week: low activity; ≥ 14 MET hours/week: moderate/high activity), which were also obtained from the questionnaires before retirement (w_−1_).

### Statistical analyses

The characteristics of the participants before retirement (w_−1_) are presented as frequencies and proportions for the categorical variables. As our aim was to detect changes between each subsequent study wave, particularly between waves before and after retirement, we identified trajectories of risky drinking using latent class analysis (LCA) 28. LCA is a statistical tool which clusters similar response profiles into distinct classes. We fitted models with one to five classes and selected a model with the best fit. Assessment of model fit was based on *P*‐values for the G^2^ statistic, Bayesian information criterion values (model with the values closest to null indicate a better fit) and the relevance of the solution for the study question. Model fit statistics for the one to five class solutions are presented in Supporting information, Table S1. The selected three‐class solution as well as those for the two‐ and four‐class solutions are presented in Supporting information, Figure S1. The three‐class solution included the following trajectories: sustained healthy drinking; temporary increase in risky drinking; and slowly declining risky drinking after retirement.

To assess pre‐retirement predictors of slowly declining risky drinking and temporary increase in risky drinking we used multinomial logistic regression models. The pre‐retirement predictors included sex, cohort, retirement age, occupational status, education, marital status, smoking, body mass index, physical activity, depression, self‐reported health, area of work‐place and job strain, all measured at the time‐point w_−1_ before retirement. Individuals with missing information on the predictor in question were not included in the analysis. The sustained healthy drinking group served as the reference category when calculating sex‐adjusted odds ratios (OR) and 95% confidence intervals (CI), but comparisons were also made between the temporary increase in risky drinking and slowly declining risky drinking groups. All analyses were performed using statistical software SAS version 9.4 (SAS Institute Inc., Cary, NC, USA). LCA models were estimated using proc lca, that included ‘covariates’ command to adjust for class uncertainty in the assessment of predictors of each trajectory. Examples of the SAS codes used are provided in the Supporting information.

## Results

**Figure 1:**
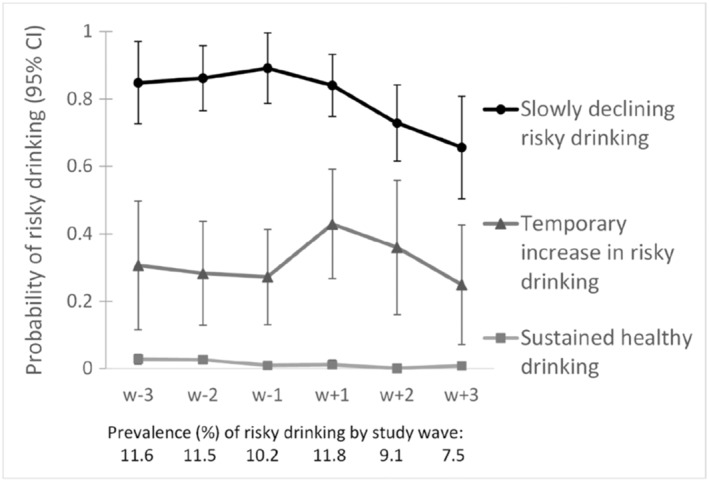
Trajectories of risky drinking over six study waves (4‐year intervals) around the time of statutory retirement

Of the 5805 participants, 1159 (20%) were men and 4646 (80%) were women. Their mean age at retirement was 61.9 years [standard deviation (SD) = 2.0]. The proportion of those reporting heavy alcohol use at w_−1_ was 7.6%, and that of those reporting at least one extreme drinking occasion was 3.3%. Prevalence of risky drinking at each wave is presented in Fig. 1.

The three latent class trajectories for risky drinking are shown in Fig. 1: the trajectory with the largest proportion of individuals (81%) indicated a group of sustained healthy drinkers with constant very low probability of risky drinking. The second largest trajectory (12% of individuals) indicated a group that had temporary increase in risky drinking around retirement transition, i.e. between time‐points w_−1_ and w_+1_. In this group, the prevalence of risky drinking was 27–31% before retirement. Following retirement, there was a sharp increase in this prevalence from 27 to 43%, which then turned into a decrease with advancing age. The third trajectory, with the smallest proportion of individuals (7%), included individuals with slowly declining risky drinking post‐retirement. In this group, the prevalence of risky drinking was 84–89% from before to after the retirement transition, after which it turned to a slight decline.

Pre‐retirement factors predicting membership of the temporary increase in the risky drinking group and the slowly declining risky drinking group adjusted for sex are presented in Table 1. The temporary increase in risky drinking around retirement group was characterized by men, current or former smokers, those reporting depression and those who had worked in the metropolitan area. These same pre‐retirement characteristics were associated with risky drinking. In addition, high occupational status and education and low physical activity characterized membership of risky drinking. Reporting job strain before retirement was protective of belonging to the risky drinking group. Compared to the slowly declining risky drinking group, the members of the group with temporary increase in risky drinking were less likely to have high socio‐economic status, be former smokers or have their work‐place in the metropolitan area (Table 1).

**Table 1:** Pre‐retirement (w_−1_) predictors for belonging to the trajectory of temporary increase in risky drinking and slowly declining risky drinking.

Variable	Participants at w_−1_	Temporary increase in risky drinking versus healthy drinking			Risky drinking versus healthy drinking			Temporary increase in risky drinking versus risky drinking		
	n (%)	OR	95% CI		OR	95% CI		OR	95% CI	
Sex										
Male versus female	1159 (20)	**2.77**	**2.16**	**3.55**	**2.66**	**2.06**	**3.42**	1.04	0.73	1.49
Cohort^a^										
2 versus 1	1579 (27)	1.05	0.79	1.40	1.13	0.85	1.52	0.93	0.60	1.43
3 versus 1	2153 (37)	1.23	0.90	1.68	0.97	0.71	1.33	1.27	0.80	2.00
Age at retirement										
60–64 versus < 60	4271 (74)	1.19	0.85	1.67	1.52	0.99	2.33	0.78	0.44	1.39
> 64 versus < 60	848 (15)	0.99	0.64	1.53	1.22	0.73	2.05	0.81	0.39	1.67
Marital status										
Single versus married/cohabiting	368 (6)	0.85	0.49	1.50	1.21	0.76	1.94	0.70	0.32	1.56
Divorced/widowed versus married/cohabiting	1107 (19)	1.15	0.86	1.53	1.16	0.86	1.57	0.99	0.64	1.52
Occupational status										
High versus low	2207 (38)	1.10	0.83	1.45	**3.15**	**2.17**	**4.57**	**0.35**	**0.22**	**0.56**
Intermediate versus low	1558 (27)	1.09	0.82	1.45	**1.56**	**1.04**	**2.34**	0.70	0.41	1.19
Education										
High versus low	3262 (56)	0.92	0.69	1.23	**1.65**	**1.12**	**2.44**	**0.56**	**0.34**	**0.91**
Intermediate versus low	1667 (29)	**0.71**	**0.51**	**0.99**	**0.61**	**0.38**	**0.99**	1.16	0.62	2.16
Self‐rated health										
Poor versus good	2126 (37)	1.06	0.85	1.33	1.14	0.89	1.44	0.93	0.66	1.32
Depression^b^										
Yes versus no	634 (12)	**1.44**	**1.05**	**1.99**	**2.20**	**1.60**	**3.01**	0.66	0.42	1.02
Smoking										
Former versus never	946 (17)	**1.85**	**1.36**	**2.52**	**3.56**	**2.73**	**4.66**	**0.52**	**0.35**	**0.78**
Current versus never	502 (9)	**3.90**	**2.70**	**5.64**	**4.81**	**3.38**	**6.83**	0.81	0.51	1.29
Physical activity										
Low versus moderate	2436 (42)	1.17	0.94	1.46	**1.30**	**1.03**	**1.64**	0.90	0.65	1.26
BMI, kg/m^2^										
25–29.9 versus < 25	2275 (41)	1.17	0.90	1.51	1.24	0.96	1.61	0.94	0.64	1.37
≥ 30 versus < 25	894 (16)	1.18	0.84	1.65	1.32	0.94	1.83	0.89	0.55	1.46
Area of work‐place										
Metropolitan versus other area	1562 (27)	**1.29**	**1.00**	**1.66**	**1.87**	**1.48**	**2.37**	**0.69**	**0.48**	**0.98**
Job strain										
yes versus no	4573 (76)	0.82	0.63	1.07	**0.59**	**0.42**	**0.81**	1.40	0.89	2.20

Bolded numbers indicate statistically significant odds ratios at the level of 0.05. Models adjusted for sex

a Categorized according to the year of the study wave before retirement (w_−1_): 1 = 2000; 2 = 2004; 3 = 2008;

b Self‐reported doctor diagnosed depression. OR = odds ratio; CI =confidence interval; BMI = body mass index.

As a post‐hoc analysis, we checked whether possible clustering of individuals within the organizations caused heterogeneity or bias to the results using a multi‐level model. The results were only marginally different, so only results from the simpler model ignoring organizations are presented.

## Discussion

The results of this longitudinal study suggest that most people maintain their healthy or risky drinking habits after retirement. However, we also observed a subgroup of people with a marked, albeit temporary, increase in risky drinking following retirement. The strongest pre‐retirement predictors for belonging to this group were being male, smoking, depressive symptoms and work‐place in the metropolitan area. These characteristics, as well as high socio‐economic status, were also associated with the trajectory of slowly declining risky drinking.

Our findings are plausible. After retirement people have more leisure time and more opportunities for different activities, and less stress 29, 30. However, retirement can also lead to reduced social control and loss of social contacts and therefore be perceived as a stressful life transition 29, 30. Both the positive and negative aspects related to changes in leisure time, stress and social networks around retirement may affect drinking behaviours although our findings suggest that, for those increasing their consumption, these changes are relatively short‐term with people returning to the pre‐retirement risky drinking habits within 4–8 years.

To our knowledge, our study is the first and largest to present patterns of risky alcohol consumption over several years around retirement. Our findings are in agreement with a previous longitudinal male‐dominated study from France, which observed an increased prevalence in heavy drinking during retirement transition followed by a decrease in the post‐retirement period, among both men and women 12. However, in that study, only average changes, rather than groups with different alcohol consumption trajectories, were assessed.

The identified predictors of the trajectory of temporary increase in risky drinking cover a wide range of factors that are generally related to higher alcohol consumption: male sex, smoking and depressive symptoms 18, 20, 21, 31. We also observed increased odds of belonging to the group of temporary increase in risky drinking in retirees whose work‐place had been in the metropolitan area. One possible explanation for this is more plentiful opportunities to engage in leisure time activities in the city than in the non‐metropolitan areas.

While higher occupational status is linked typically to a more favourable health profile 17, 32, people from higher occupational status groups have reported higher alcohol consumption than those from lower occupational status groups in cohort studies of public sector employees 17. In the present study, higher occupational status and education were associated with increased odds of belonging to the trajectory of slowly declining risky drinking. High socio‐economic status, along with being a former smoker and having worked in the metropolitan area, were characteristics that best distinguished the slowly declining risky drinking group from the group with a temporary increase in risky drinking.

The reasons for the decreasing trajectories of risky drinking in the post‐retirement period—observed in both ours and the French study—are not known. It is possible that decreased risky drinking is linked to age‐related deterioration of health status 33. Health‐related sample attrition is one possible explanation for the observed decrease in consumption, if it was only those with the healthiest behaviours who had responded to the study questionnaires after retirement. We consider this an unlikely explanation for our findings because, of all the statutory retirees in our data set, only 0.3% of those reporting risky drinking and 0.7% of those reporting poor self‐rated health before retirement (w_−1_) did not provide information regarding alcohol consumption after retirement (w_+1_).

Our study demonstrates the importance of using multiple measurements of alcohol consumption both before and after retirement. Previous studies, typically using information from a single time‐point before and after retirement, have reported mixed findings, suggesting either an increase or no change in alcohol consumption 6, 7. This is unsurprising, as such a design cannot distinguish temporary change in consumption around the retirement transition from the long‐term trends in alcohol use at older ages. Differences in study populations, study contexts and the cultures of alcohol consumption, in addition to the varying model adjustments and definitions of retirement, are likely to further increase heterogeneity between studies 4. There is a need for future studies to identify other life changes, such as becoming unemployed, that may drive the temporary increase in risky drinking, as well as studies to determine whether such an increase predicts risky drinking later in life, or is likely to have longer‐term consequences on health. In practice, occupational health services and the employers could develop strategies to make the life transition for retiring employees smoother and healthier.

Our findings are likely to be conservative, as we included only participants who entered statutory retirement as their first retirement. Our study population did not include those with the greatest number of health problems, who most probably retire on health grounds, potentially reducing the variation in health behaviours that correlate with heavy alcohol use 34. As all participants were entering statutory retirement within an 11‐year time‐period, they most probably belonged to the same generation. Thus, birth cohort effects were not a source of major bias in this study.

There are some limitations to our study that need to be considered when interpreting the findings. One limitation is that self‐reported alcohol consumption is prone to recall and information bias, possibly resulting in under‐reported consumption levels 35. However, our focus was on trajectories of risky drinking rather than levels of alcohol consumption, and there is no reason to assume that these biases would be different for the different study waves. Most of the studied predictors were assessed using self‐reports, including body mass index (BMI), which may have resulted in under‐ or over‐estimations of some associations. It is also possible that personality traits (e.g. extraversion, openness to experience or neuroticism) or the working status of the partner may affect the relationship between transition to retirement and risky drinking, but no additional data were available to account for the possible moderating effect of these factors. Although the latent class analysis identifies a certain number of discrete latent trajectories in observed data, it cannot prove that such discrete trajectories actually exist and remain as observed over time. Our study population consisted of relatively healthy public sector employees from Finland who transitioned from full‐time work into statutory retirement. Due to the nature of public sector jobs, women were over‐represented in this cohort. Thus, the generalizability of the findings to other working sectors, retirement types, cohorts and other countries and cultures may be limited. Indeed, drinking behaviours and cultural norms are known to vary between countries, such as Finland, the United Kingdom, Australia and the United States 36. The findings may also vary according to which birth cohorts and time‐‐periods (e.g. whether new regulations regarding alcohol resale took place) are under study.

## Conclusions

We identified three trajectories of risky alcohol consumption around the time of statutory retirement among public sector employees: sustained healthy drinking, slowly declining risky drinking after retirement and temporary increase in risky drinking in retirement transition. Belonging to the trajectory of temporarily increased risky drinking around retirement was predicted by common risk factors for heavy drinking such as being male, smoking and depressive symptoms, but also health‐neutral factors (pre‐retirement work‐place in the metropolitan area). Further research is needed to evaluate how generalizable our findings are, and whether interventions targeted to groups characterized by these factors can prevent risky drinking and thus benefit ageing employees during their retirement transition.

### Declaration of interests

None.

## Supporting information


**Figure S1** Trajectories for three differenct solutions with varying number of latent classes.
**Table S1** Model fit statistics of the latent class analysis (LCA) models with different 1–5 latent classes.

## References

[1] Gbd 2013 Risk Factors Collaborators , Forouzanfar M. H. , Alexander L. , Anderson H. R. , Bachman V. F. , Biryukov S. *et al.* Global, regional, and national comparative risk assessment of 79 behavioural, environmental and occupational, and metabolic risks or clusters of risks in 188 countries, 1990–2013: a systematic analysis for the global burden of disease study 2013. Lancet 2015; 386: 2287–2323. 26364544 10.1016/S0140-6736(15)00128-2 PMC4685753

[2] Reczek C. , Pudrovska T. , Carr D. , Thomeer M. B. , Umberson D. Marital histories and heavy alcohol use among older adults. J Health Soc Behav 2016; 57: 77–96. 26957135 10.1177/0022146515628028 PMC4785832

[3] Bosque‐Prous M. , Espelt A. , Sordo L. , Guitart A. M. , Brugal M. T. , Bravo M. J. Job loss, unemployment and the incidence of hazardous drinking during the late 2000s recession in Europe among adults aged 50–64 years. PLOS ONE 2015; 10: e0140017. 26445239 10.1371/journal.pone.0140017 PMC4596847

[4] Kuerbis A. , Sacco P. The impact of retirement on the drinking patterns of older adults: a review. Addict Behav 2012; 37: 587–595. 22365490 10.1016/j.addbeh.2012.01.022

[5] Perreira K. M. , Sloan F. A. Life events and alcohol consumption among mature adults: a longitudinal analysis. J Stud Alcohol 2001; 62: 501–508. 11513228 10.15288/jsa.2001.62.501

[6] Henkens K. , Van Solinge H. , Gallo W. T. Effects of retirement voluntariness on changes in smoking, drinking and physical activity among Dutch older workers. Eur J Public Health 2008; 18: 644–649. 18927184 10.1093/eurpub/ckn095 PMC2727140

[7] Ding D. , Grunseit A. C. , Chau J. Y. , Vo K. , Byles J. , Bauman A. E. Retirement—a transition to a healthier lifestyle?: evidence from a large Australian study. Am J Prev Med 2016; 51: 170–178. 26972491 10.1016/j.amepre.2016.01.019

[8] Syse A. , Veenstra M. , Furunes T. , Mykletun R. J. , Solem P. E. Changes in health and health behavior associated with retirement. J Aging Health 2017; 29: 99–127. 26721789 10.1177/0898264315624906

[9] Bobo J. K. , Greek A. A. , Klepinger D. H. , Herting J. R. Predicting 10‐year alcohol use trajectories among men age 50 years and older. Am J Geriatr Psychiatry 2013; 21: 204–213. 23343494 10.1016/j.jagp.2012.10.021

[10] Bobo J. K. , Greek A. A. Increasing and decreasing alcohol use trajectories among older women in the U.S. across a 10‐year interval. Int J Environ Res Public Health 2011; 8: 3263–3276. 21909305 10.3390/ijerph8083263 PMC3166741

[11] Collins L. M. , Lanza S. T. Latent Class and Latent Transition Analysis. Hoboken, NJ: John Wiley & Sons, Inc.; 2010.

[12] Zins M. , Gueguen A. , Kivimaki M. , Singh‐Manoux A. , Leclerc A. , Vahtera J. *et al.* Effect of retirement on alcohol consumption: longitudinal evidence from the French Gazel cohort study. PLOS ONE 2011; 6: e26531. 22028898 10.1371/journal.pone.0026531 PMC3197660

[13] Kivimaki M. , Hamer M. , Batty G. D. , Geddes J. R. , Tabak A. G. , Pentti J. *et al.* Antidepressant medication use, weight gain, and risk of type 2 diabetes: a population‐based study. Diabetes Care 2010; 33: 2611–2616. 20823343 10.2337/dc10-1187 PMC2992199

[14] Finnish Center for Pensions . The role of the Finnish Centre for Pensions in the earnings‐related pension scheme; 2016 Available at: http://www.etk.fi/en/the‐pension‐system‐2/the‐pension‐system/administration‐and‐supervision/parties‐to‐pension‐scheme/finnish‐centre‐for‐pensions/ (accessed 13 December 2016) (Archived at http://www.webcitation.org/6pCbeBSUx on 24 March 2017).

[15] Halonen J. I. , Kivimaki M. , Pentti J. , Virtanen M. , Subramanian S. V. , Kawachi I. *et al.* Association of the availability of beer, wine, and liquor outlets with beverage‐specific alcohol consumption: a cohort study. Alcohol Clin Exp Res 2014; 38: 1086–1093. 24460841 10.1111/acer.12350

[16] Työterveyslaitos Ja Sosiaali‐Ja Terveysministeriö [Finnish Institute of Occupatinal Health and Finnish Ministry of Social Affairs and Health] . Riskikulutuksen varhainen tunnistaminen ja mini‐interventio‐hoitosuosituksen yhteenveto [adapted translation into Finnish based on: Anderson P., Gual A., Colom J. (2005). Alcohol and Primary Health Care: Clinical Guidelines on Identification and Brief Interventions. Department of Health of the Government of Catalonia: Barcelona]; 2006.

[17] Stringhini S. , Dugravot A. , Shipley M. , Goldberg M. , Zins M. , Kivimaki M. *et al.* Health behaviours, socioeconomic status, and mortality: further analyses of the British Whitehall II and the French GAZEL prospective cohorts. PLOS Med 2011; 8: e1000419. 21364974 10.1371/journal.pmed.1000419 PMC3043001

[18] Karlamangla A. , Zhou K. , Reuben D. , Greendale G. , Moore A. Longitudinal trajectories of heavy drinking in adults in the United States of America. Addiction 2006; 101: 91–99. 16393195 10.1111/j.1360-0443.2005.01299.x

[19] Paljarvi T. , Makela P. , Poikolainen K. , Suominen S. , Car J. , Koskenvuo M. Subjective measures of binge drinking and alcohol‐specific adverse health outcomes: a prospective cohort study. Addiction 2012; 107: 323–330. 21801266 10.1111/j.1360-0443.2011.03596.x

[20] Härkönen J. T. , Mäkelä P. Age, period and cohort analysis of light and binge drinking in Finland, 1968–2008. Alcohol Alcohol 2011; 46: 349–356. 21508197 10.1093/alcalc/agr025

[21] Moore A. A. , Gould R. , Reuben D. B. , Greendale G. A. , Carter M. K. , Zhou K. *et al.* Longitudinal patterns and predictors of alcohol consumption in the United States. Am J Public Health 2005; 95: 458–465. 15727977 10.2105/AJPH.2003.019471 PMC1449202

[22] Statistics Finland . Classification of Occupations 2001; 2002 Available at: http://www.stat.fi/meta/luokitukset/ammatti/001‐2001/index_en.html (accessed 13 December 2016) (Archived at http://www.webcitation.org/6pCc64Vuz on 24 March 2017).

[23] Halonen J. I. , Kivimaki M. , Virtanen M. , Pentti J. , Subramanian S. V. , Kawachi I. *et al.* Living in proximity of a bar and risky alcohol behaviours: a longitudinal study. Addiction 2013; 108: 320–328. 22897634 10.1111/j.1360-0443.2012.04053.x PMC3529803

[24] Stenholm S. , Pulakka A. , Kawachi I. , Oksanen T. , Halonen J. I. , Aalto V. *et al.* Changes in physical activity during transition to retirement: a cohort study. Int J Behav Nutr Phys Act 2016; 13: 51. 27084334 10.1186/s12966-016-0375-9 PMC4833915

[25] Statistics Finland . Employment; 2011 Available at: http://www.stat.fi/meta/til/tyokay_en.html (accessed 13 December 2016) (Archived at http://www.webcitation.org/6pCcHGfKr on 24 March 2017).

[26] World Health Organization (WHO) . Obesity: preventing and managing the global epidemic. Report of a WHO consultation. World Health Organ Tech Rep Ser 2000; 894: i–xii, 1–253. 11234459

[27] Ainsworth B. E. , Haskell W. L. , Herrmann S. D. , Meckes N. , Bassett D. R. Jr. , Tudor‐Locke C. *et al.* Compendium of physical activities: a second update of codes and MET values. Med Sci Sports Exerc 2011; 2011: 1575–1581. 10.1249/MSS.0b013e31821ece12 21681120

[28] Lanza S. T. , Collins L. M. , Lemmon D. R. , Schafer J. L. PROC LCA: a SAS procedure for latent class analysis. Struct Equ Model Multidiscip J 2007; 14: 671–694. 10.1080/10705510701575602 PMC2785099 19953201

[29] Gallo W. The association of retirement with physical and behavioural health In: Wang M., editor. The Oxford Handbook of Retirement. Oxford, UK: Oxford University Press; 2013.

[30] van der Heide I. , van R. M. , Robroek S. J. , Burdorf A. , Proper K. I. Is retirement good for your health? A systematic review of longitudinal studies. BMC Public Health 2013; 13: 1180. 24330730 10.1186/1471-2458-13-1180 PMC4029767

[31] Pardini D. , White H. R. , Stouthamer‐Loeber M. Early adolescent psychopathology as a predictor of alcohol use disorders by young adulthood. Drug Alcohol Depend 2007; 88: S38–S49. 17257781 10.1016/j.drugalcdep.2006.12.014 PMC2034413

[32] Mackenbach J. P. , Stirbu I. , Roskam A. J. , Schaap M. M. , Menvielle G. , Leinsalu M. *et al.* Socioeconomic inequalities in health in 22 European countries. N Engl J Med 2008; 358: 2468–2481. 18525043 10.1056/NEJMsa0707519

[33] Holdsworth C. , Mendonca M. , Pikhart H. , Frisher M. , De C. , Shelton N. Is regular drinking in later life an indicator of good health? Evidence from the English longitudinal study of ageing. J Epidemiol Community Health 2016; 70: 764–770. 26797821 10.1136/jech-2015-206949 PMC4975801

[34] Korhonen T. , Smeds E. , Silventoinen K. , Heikkila K. , Kaprio J. Cigarette smoking and alcohol use as predictors of disability retirement: a population‐based cohort study. Drug Alcohol Depend 2015; 155: 260–266. 26305074 10.1016/j.drugalcdep.2015.06.047

[35] Ekholm O. , Strandberg‐Larsen K. , Gronbaek M. Influence of the recall period on a beverage‐specific weekly drinking measure for alcohol intake. Eur J Clin Nutr 2011; 65: 520–525. 21326272 10.1038/ejcn.2011.1

[36] World Health Organization (WHO) . WHO Global Status Report on Alcohol and Health 2014—Individual Country Profiles. Geneva: WHO; 2016.

