# Supplementary material for: Trajectories of risky drinking around the time of statutory retirement: a longitudinal latent class analysis
Source: Addiction. 2017 Apr 16;112(7):1163–70. doi: 10.1111/add.13811 (PMC5498817; doi:10.1111/add.13811)
Supplement: Supplementary file 1 — Figure S1 Trajectories for three differenct solutions with varying number of latent classes. Table S1 Model fit statistics of the latent class analysis (LCA) models with different 1–5 latent classes. [file ADD-112-1163-s001.docx]

**Supporting information**

**Trajectories of risky drinking around the time of statutory retirement: a longitudinal latent class analysis**

Jaana I. Halonen, Sari Stenholm, Anna Pulakka, Ichiro Kawachi, Ville Aalto, Jaana Pentti, Tea Lallukka, Marianna Virtanen, Jussi Vahtera, Mika Kivimäki

Examples of the SAS codes used for the analyses

1. SAS code for latent class trajectory analysis of risky drinking (Figure 1)

proc lca data=e1 outpost=post_best outparam=lca_param;

id id;

nclass 3;

items risky1-risky6;

categories 2 2 2 2 2 2;

rho prior=1;

seed 1957;

run;

** Average posterior probabilities **;

data post_best2;

set post_best;

if best=1 then postprob=POSTLC1;

if best=2 then postprob=POSTLC2;

if best=3 then postprob=POSTLC3;

latent=best;

keep id latent postprob;

proc means data=post_best2 fw=5;

var postprob;

class latent;

run;

1. SAS code for latent class trajectory analysis using multinomial logistic regression analysis to assess covariates that predict membership of each trajectory (Table 1)

** Covariates **;

proc lca data=e1 start=lca_param;

id id; nclass 3; items risky1-risky6; categories 2 2 2 2 2 2; rho prior=1;

covariate sex marital;

reference 3;

proc lca data=e1 start=lca_param;

id id; nclass 3; items risky1-risky6; categories 2 2 2 2 2 2; rho prior=1;

covariate sex marital;

reference 2;

run;

**Table S1**. Model fit statistics of the latent class analysis (LCA) models with different one to five latent classes.

|  | Number of latent classes | | | | |
| --- | --- | --- | --- | --- | --- |
| Fit statistic | 1 | 2 | 3 | 4 | 5 |
| Log-likelihood: | -7262.1 | -5571.9 | -5553.3 | -5527.8 | -5526.4 |
| G^2^: | 3472.3 | 91.9 | 54.7 | 3.7 | 0.9 |
| Degrees of freedom: | 57 | 50 | 43 | 36 | 29 |
| p (G^2^) | <.001 | <0.001 | 0.109 | 1.000 | 1.000 |
| AIC^*^: | 3484.3 | 117.9 | 94.7 | 57.7 | 68.9 |
| BIC^†^: | 3524.3 | 204.5 | 228.0 | 237.7 | 295.5 |
| Entropy: | 1 | 0.91 | 0.79 | 0.82 | 0.83 |
| Average posterior probabilities | 1 | 0.98/0.95 | 0.69/0.83/ 0.96 | 0.65/0.82/ 0.95/0.64 | 0.55/0.83/ 0.60/0.63/ 0.96 |

^*^ AIC = Akaike’s Information Criterion
^†^ BIC = Bayesian Information Criterion

**
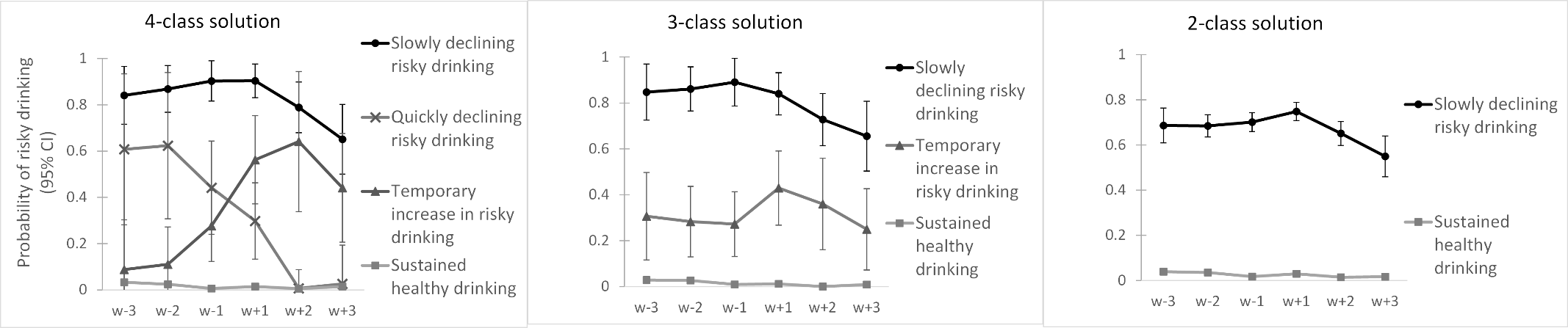
**

**Fig S1.** Trajectories for three differenct solutions with varying number of latent classes.
